# An exploration of GuaLouQuMaiWan related novel prognostic model and search for potential compound pachymic acid in bladder cancer

**DOI:** 10.3389/fonc.2026.1714939

**Published:** 2026-04-15

**Authors:** Qiliang Teng, Shumin Cheng, Jia Chen, Hongya Zhu, Kai Hu, Lingling Sun, Jin Zhao, Zhenbo Yuan, Wenjing Wang, Li Zhang, Taoli Liu, Jiahao Feng

**Affiliations:** 1 Department of Urology, The Seventh Affiliated Hospital of Sun Yat-sen University, Shenzhen, China; 2 Scientific Research Center Department, The Seventh Affiliated Hospital of Sun Yat-sen University, Shenzhen, China; 3 Digestive Disease Center, The Seventh Affiliated Hospital of Sun Yat-sen University, Shenzhen, China; 4 School of Medicine, Sun Yat-sen University, Shenzhen, China; 5 Traditional Chinese Medicine Department, The Seventh Affiliated Hospital of Sun Yat-sen University, Shenzhen, China; 6 Shenzhen Key Laboratory of Chinese Medicine Active substance screening and Translational Research, Shenzhen, China

**Keywords:** bladder cancer (BC), EGFR, FABP6, GuaLouQuMaiWan (GLQMW), pachymic acid (PA)

## Abstract

**Objective:**

Bladder cancer (BC) is a highly prevalent malignant tumor. The traditional Chinese medicine formula Guo Lou Qu Mai Wan (GLQMW), when used in conjunction with chemotherapy, has been shown to reduce adverse reactions and prolong survival time, although its specific mechanisms remain unclear. This study aims to investigate whether GLQMW exerts its therapeutic effects on bladder cancer by modulating the tumor immune microenvironment.

**Methods:**

To elucidate the molecular mechanisms of GLQMW, we employed bioinformatics analysis, systems pharmacology, and molecular docking to explore the prognostic value of GLQMW-related target genes for BC and to establish a prognostic prediction model. We analyzed the relationship between GLQMW-related target genes and immune cell infiltration, as well as the compositional differences of immune cell subpopulations across different risk groups. Molecular docking was used to screen for pachymic acid (PA) as the main anticancer active compound, and CCK8 and western blot were used to determine the target of PA as the active compound.

**Result:**

In our immune infiltration analysis, the expression levels of five genes (MAPK12, MAN1B1, EGFR, FABP6, and ZAP70) were found to be associated with immune cells. Moreover, a higher presence of naïve B cells, plasma cells, CD8+ T cells, and Tregs was observed in the low-risk group, indicating that GLQMW can significantly impact the immune microenvironment by targeting these five model genes, thereby exerting therapeutic effects. In the single-cell data analysis, our results demonstrated that the percentage of CD8 T+ cells and plasma cells in tumor tissue was significantly lower than that in adjacent non-tumor tissue. In addition, through drug similarity analysis and molecular docking, we identified PA as a potential anti-tumor compound. Furthermore, PA was validated *in vitro* to upregulate FABP6 and downregulate EGFR expression and suppress the bladder cancer progression *in vivo*.

**Conclusions:**

PA, the active ingredient of GLQMW, can inhibit BC by inhibiting EGFR and upregulating FABP6.

## 1 Introduction

Bladder cancer (BC) is the second most common urological malignant disease, affecting more than 500,000 patients per year globally, the incidence of which is increasing with age and is almost three times as many men as women (1, 2). The development of BC is associated with risk factors, including genetic abnormalities, environmental exposures and other chronic diseases of urinary system (3). Its clinical presentation is composed of non-muscle-invasive BC (NMIBC), muscle-invasive BC (MIBC) and metastatic urothelial cancer, which hematuria as the major presenting symptom, accounting for roughly 75% patients in NMIBC and 25% patients in MIBC (4). At present, the mainstay of treatment for patients with NMIBC is mainly transurethral resection of bladder tumor combined with bladder perfusion, while MIBC and metastatic urothelial require surgical removal of all or part of the urinary bladder (1). Moreover, bladder-sparing trimodality treatment is used to the treatment of patients (5). Nonetheless, several studies reported that there is still a high rate of recurrence in NMIBC and MIBC, and patients with metastatic bladder cancer have a high risk of death because of rapid progress of tumor (6, 7). Clinically, the combination of gemcitabine and cisplatin (GC) with immunotherapy is a common choice (8). However, this approach often leads to drug resistance or suboptimal initial efficacy, causing BC patients to lose treatment opportunities.

The mechanisms underlying bladder cancer cell resistance or the failure of immunotherapy are not yet fully understood. Current studies indicates that bladder cancer can develop GC resistance by activating various pathways, including PI3K/AKT (9, 10), circPTK2/PABPC1/SETDB1 (11), LKB1-AMPK-LC3 (12), and HIF-1α (13). Furthermore, insufficient CD8 cell infiltration and a non-inflammatory tumor microenvironment (TME) can contribute to the failure of immunotherapy (14, 15). Therefore, to improve survival rates for BC patients, there is an urgent need to explore new combination therapies. The tumor microenvironment is a critical factor influencing tumor development and prognosis. Existing studies suggest that enhancing immune infiltration in tumors can improve treatment efficacy when combined with chemotherapy (16). This presents a promising direction for the development of novel combination therapies. However, this approach often leads to drug resistance. Therefore, to improve survival rates for bladder cancer patients, there is an urgent need to explore new combination therapies. The tumor microenvironment is a critical factor influencing tumor development and prognosis. Existing studies suggest that enhancing immune infiltration in tumors can improve treatment efficacy when combined with chemotherapy. This is a promising direction for the development of novel combination therapies.

Multiple lines of evidence suggest that a subset of genes—including MAPK12, ZAP70, MAN1B1, EGFR, and FABP6—may contribute to prognosis or immune microenvironment through distinct mechanisms. In multi-cohort prognostic screening using TCGA and GEO datasets, MAPK12 emerged as one of the risk genes within an 11-gene Cox signature built by univariate Cox and LASSO regression; this model consistently stratified patients into high- and low-risk groups across independent cohorts, with poorer outcomes in the high-risk group and acceptable 1-year predictive performance (AUC > 0.65) (17). Similarly, ZAP70 was identified as a protective factor in an immune-related prognostic model developed in TCGA and validated in external GEO cohorts, suggesting that higher ZAP70 expression correlates with improved overall survival (18). In contrast, MAN1B1 has more direct mechanistic and experimental support linking it to immune evasion in bladder cancer. Specifically, aberrant glycosylation of CD47 was reported to strengthen CD47 interaction with SIRP-α, thereby enhancing the “don’t-eat-me” signal and suppressing phagocytosis. MAN1B1, a glycosylation-related enzyme, was shown to be highly expressed in bladder cancer and responsible for CD47 glycosylation. Upstream, abnormal ERK activation stabilized MAN1B1 by regulating its interaction with the E3 ubiquitin ligase HRD1, thereby promoting CD47 glycosylation and immune escape. Importantly, both *in vitro* and *in vivo* experiments demonstrated that MAN1B1 knockout attenuated CD47-mediated anti-phagocytosis, and pharmacologic MAN1B1 inhibition promoted phagocytosis without inducing anemia, highlighting a potential immunotherapeutic strategy that may avoid a key limitation of direct anti-CD47 approaches (19). EGFR also appears clinically relevant in a molecularly defined subgroup of bladder cancer. Recent findings indicate that EGFR pathway activation characterizes a subset of basal-like muscle-invasive bladder cancers (approximately 23.5% of MIBC), where EGFR overexpression is associated with poor prognosis and tumor cells bearing this signature display sensitivity to EGFR inhibitors (20). Additionally, FABP6 has been functionally implicated in bladder cancer growth, motility, and therapy response. Knockdown of FABP6 reduced proliferation in both low-grade and high-grade bladder cancer cell lines, accompanied by cell-cycle blockade and reduced expression of CDK2, CDK4, and Ki67. FABP6 silencing also downregulated autophagy markers and activated AKT–mTOR signaling, and additional PI3K/AKT inhibition further decreased cell viability, suggesting pathway interplay that may be therapeutically exploitable. Moreover, FABP6 knockdown suppressed migration with reduction of focal adhesion complexes, and in a xenograft model, shFABP6 combined with cisplatin more effectively inhibited tumor growth, supporting FABP6 as a potential target for restraining progression and enhancing chemosensitivity (21).

GuaLouQuMaiWan (GLQMW), a classical traditional Chinese medicine (TCM) formula, deriving from “Jin Kui Yaolve” written by Zhang Zhongjing which is a famous physician from the Eastern Han Dynasty of China. GLQMW is formulated of five herbs, including Fuzi (*Aconiti Lateralis Radix Praeparata*), Fulin (*Poria Cocos (Schw.) Wolf.*), Qumai (*Dianthi Herba*), Shanyao (*Rhizoma Dioscoreae*), and Tianhuafen (*Trichosanthis Radix*) (22). According to the therapeutic principle of TCM, this formula can invigorate the spleen and kidney, eliminate dampness and phlegm and diuresis, which has been applied in clinical practice to treat diseases associated with urinary system, such as oligospermia, prostatitis and urethritis syndrome in China (23). Of note, in TCM, tumors are thought to be formed by mutually obstructed of heat, phlegm and stasis, so the bladder cancer is mainly caused by the accumulation of heat, phlegm and stasis. The “kidney channel” is connected with the “bladder channel”, which have an exterior-interior relationship and influence each other (13), so the TCM treatment of bladder cancer takes the kidney as the starting point.

We summarize the effective compounds of GLQMW in the TCMSP, because it collects the basic physical and chemical properties of herbs and related compounds contained in the Chinese Pharmacopoeia (24). The PharmMapper server (25) is used to predict the potential target of compounds. Our research uses network pharmacology (26) and bioinformatics (27) to analyze drugs and disease risk indicators to obtain the main targets and compounds, and combines GLQMW drug targets with the prognosis risk model of bladder cancer to jointly analyze the therapeutic mechanism of GLQMW. At the same time, we used Python to run RDKit to analyze the similarity of small molecular compounds. Based on the results from RDKit, we hypothesize that GLQMW may contain compounds that inhibit the growth of bladder cancer and affect its drug resistance and prognosis. Since the interactions and binding between molecules depend on their chemical structures, we utilized methods such as transcriptomic analysis, network pharmacology, molecular docking, and single-cell analysis to identify the targets through which GLQMW exerts its anti-bladder cancer effects.

## 2 Method

### 2.1 Screening of active compounds and potential targets for GLQMW

The active compounds of GLQMW were retrieved from Traditional Chinese Medicine Database and Analysis Platform (TCMSP, https://tcmsp-e.com/), while those active compounds were retained for subsequent analysis based on oral bioavailability (OB) ≥ 30%, drug-likeness (DL) ≥ 0.18 and Caco ≥ -0.04 (28). The target proteins corresponding to the active components were obtained by target information function in TCMSP database. Finally, UniProt database was utilized to annotate the resulting target protein names (29).

### 2.2 Raw data

Level 3 TCGA RNA-seq data of bladder cancer (including 19 normal and 411 tumor samples) and the corresponding clinical data were downloaded from TCGA dataset. By filtering out the normal paracancer tissue, the expression data for 411 cancer tissues were kept for downstream analysis, and 406 samples of which have available survival information. In addition, three separate bladder cancer cohort used in this study as validation datasets (GSE13507, GSE31684 and GSE32894) were downloaded from the GEO database (https://www.ncbi.nlm.nih.gov/geo). For multiple datasets, use “sva” and “limma” package to remove batch effects of merging datasets, and present boxplot of the data before and after processing. Compounds of GLQMW and its’ target protein origin from TCMSP (https://www.tcmsp-e.com/) and PharmMapper server, respectively.

### 2.3 Consensus clustering

Consensus clustering was introduced for classifying the BC patients into different subgroup. The K‐means algorithm with the Spearman distance was used for clustering. The cluster number was set to a range (1–10).

### 2.4 Survival curves

The relationship between kinds of scores and survival was explored by plotting the Kaplan–Meier curve using R package ‘survival’ and ‘survminer’. Log Rank test was used to test the differences of OS between defined high and low groups.

### 2.5 Differential gene expression analysis

Differential expression analysis was conducted using the R package ‘DESeq2’. The screening conditions for the differentially expressed genes (DEGs) were: |log2FoldChange| >1.5, padj <0.05. Heatmaps of differential genes were drawn using the R-package ‘pheatmap’. For subclass-specific genes, only genes with significant differences in expression (|log2FC| >1.5, padj <0.05) in all three possible comparisons were considered subclass-specific genes.

### 2.6 GO and KEGG enrichment analysis, ssGSEA analysis and tumor infiltration immune cells profile

GO and KEGG enrichment analyses were performed with the aid of R packages ‘clusterProfiler’, ‘enrichplot’, and ‘ggplot2’. Only terms with both p- and q-value of <0.05 were considered significantly enriched. ssGSEA analysis was conducted using R package ‘GSVA’. Hallmark geneset was downloaded from the Molecular Signatures Database (MSigDB).

In combination with the LM22 signature matrix, normalized gene expression data (FPKM) were used to calculate the relative proportions of 22 types of infiltrating immune cells via CIBERSORT algorithm.

### 2.7 Establishment of the five-gene risk prediction model

We summarized the therapeutic target of GLQMW as GLQMW-related gene set, and assessed the relationships between the expression levels of the selected GLQMW-related genes and overall survival of patients by univariate Cox regression analysis. The significant genes with p <0.05 were screened out for further analysis. We randomly divided the data from TCGA cohort as internal train set and internal validation set (1:1), the entire cohort as test cohort (n = 406). Subsequently, using R package ‘glmnet’, we performed the Least Absolute Shrinkage and Selector Operation (LASSO) analysis that could reduce the estimation variance while providing an explicable final model. We selected the λ with the least cross-validation error and identified the key genes affecting patients’ prognosis. Finally, multivariate Cox regression analysis was conducted to construct the TME-based signature for predicting the prognosis in BC patients. The time-dependent ROC curve performed by R package ‘survivalROC’ and Kaplan–Meier survival curve analysis performed by R package ‘survival’ were employed to verify the accuracy of the prognostic value of the five-gene signature.

### 2.8 Construction of the protein-protein interaction network

The intersection targets of GLQMW and BC were input to the STRING platform to construct the PPI network by selecting the species as Homo sapiens, the minimum required interaction score as 0.40, and removing the disconnect target protein nodes from PPI network (30). Then, the result of PPI network was saved as a TSV format file and imported into Cytoscape (version 3.9.1) for network topology analysis and visualization.

### 2.9 Construction of the “active component-target” network

To further clarify the mechanism of GLQMW on BC, the active compounds-target network was constructed by using Cytoscape. In these network, the intersection of the relevant targets for each compounds in GLQMW and related targets in BC were expressed as nodes and their mutual relationship were expressed as edges.

### 2.10 Molecular docking

Molecular docking was perform to verify the binding of the thirteen active components of GLQMW and five target proteins using AutoDock Tool (version 4.2.6). The 3D structure of components and target proteins was acquired from TCMSP and Protein Data Bank (PDB) database. Moreover, Finally, PyMOL software (version 2.6) were used to visualize analysis and calculate their RMSD scores.

### 2.11 Predicting combination medication

Divide the risk into high-risk and low-risk groups based on five gene characteristics, and conduct differential expression analysis again. Input the DEGs obtained into the Connectivity Map (CMap) based on upregulated- and downregulated- DEGs, and obtain drugs related to the expression profile. Based on the ranking of the output small molecule drugs (the larger score, the more likely it is for the low-risk group to progress towards the high-risk group, while the lower score, the more likely it is to reverse the progression), the top drugs with negative scores are sorted for functionality and selected for drugs that can be combined with GLQMW.

### 2.12 Drug similarity analysis

Download all drug information from DrugBank (https://go.drugbank.com/), filtering to retain detailed information on all small molecule drugs while removing irrelevant protein drug information. Use Python to run RDKit, converting the small molecule structural information from GLQMW into compound fingerprints for comparison of compound similarity. By comparing the drugs in GLQMW with those recorded in DrugBank, we will filter out drugs similar to the small molecules in GLQMW using a threshold of 0.6.

### 2.13 Pan-cancer analysis

We used TIMER to analyze the expression of MAPK12, MAN1B1, EGFR, FABP6, and ZAP70 in BLCA, KIRC, and PRAD. We further analyzed their correlation with immune cells, including B cells, CD8+ T cells, CD4+ T cells, macrophages, neutrophils, and dendritic cells. TIMER2 was used to evaluate the differential expression of MAPK12, MAN1B1, EGFR, FABP6, and ZAP70 between tumor and adjacent normal tissues in the TCGA database.

### 2.14 Single cell analysis

We collected GSE149652 from the GEO database, representing respectively a dataset of bladder cancer samples treated with PD-L1. Single-cell RNA sequencing data were analyzed using the R package Seurat (31). Quality control measures were implemented, excluding cells with fewer than 200 or more than 4000 gene expressions, total UMI counts exceeding 15000, mitochondrial gene expression exceeding 10%, hemoglobin gene expression exceeding 1%, and ribosomal gene expression exceeding 50%. Ultimately, 90,303 cells were retained for subsequent analysis. The LogNormalize method ensured data normalization, and the R package Harmony was used to remove batch effects (32). The FindVariableFeatures function identified the top 2000 variable genes. Principal component analysis, t-SNE, and UMAP were conducted for dimensionality reduction, followed by cell clustering using the FindNeighbors and FindClusters functions. Cell annotation was achieved using the R package SingleR (33).

### 2.15 Cell culture and treatment

T24 cells and MB49 cells were cultured in 1640 medium (gibcol) and DMEM respectively, supplemented with 10%fbs+1% double antibody, 37°C, 5% carbon dioxide. Passage was digested with pancreatin (25200072, GIBCO) and passaged 1/4 each time. When the cells grew to 70%, they were treated with drug concentration gradient (0, 1, 2, 5, 10, 20, 40, 80) for 24h, and the cell survival rate was detected using CCK8 kit.

### 2.16 Western blot and reverse transcription polymerase chain reaction

Briefly, T24 and MB49 cells were lysed using RIPA buffer containing a complete protease inhibitor cocktail (Roche). Protein concentrations were determined using a BCA protein assay kit (Vazyme). Proteins were then electrophoresed in SDS–PAGE and transferred on a PVDF membrane(Merck millipore). After incubation in 5% non-fat milk, membranes were immunoblotted with the following antibodies: FABP6 (Abcam, human), EGFR (Cell Signaling Technology, human), GAPDH (Abcam, human).

RNA extraction: Total RNA was extracted using RNA Extraction Kit (Vazyme) and stored at -80°C. Total RNA was used to synthesize cDNA using a reverse transcription Kit (Vazyme); Subsequently, the real-time PCR amplification reaction (RT qPCR) was performed in a 96 well reaction plate (thermo) with a reaction volume of 20 µ L.

### 2.17 Plate cloning and invasion experiments

Cells (1000 cells/well) were seeded into a 6-well plate and cultured at 37 °C with 5% CO2 for 16 hours. Afterward, pachymic acid was added, and the cells were further cultured in the incubator. The medium was changed every 3 days. After 10 days of culture, the cells were fixed with tissue fixative for 20 minutes and then stained with 0.5% crystal violet solution for 20 minutes. The number of colonies was counted using Image J software. The experiment was conducted in triplicate per group.

Using Transwell chambers, a layer of matrix gel was first applied to the upper chamber. After the gel had solidified, 200 µL of medium containing 2 × 10^5 cells was added to the upper chamber, followed by incubation at 37 °C with 5% CO2 for 48 hours. After the incubation period, the cells were fixed with 4% paraformaldehyde and then stained with 0.5% crystal violet solution. Finally, the cells that had invaded from the upper chamber to the lower chamber were observed using an inverted microscope, and quantitative analysis was performed using Image J software.

### 2.18 *In vivo* xenograft tumor-growth experiments

To generate the xenograft bladder cancer model, we implanted 2 × 10^5^ MB49 cells mixed in 1 × PBS into the right flanks of C57BL/6 mice. When the tumors reached more than 4–5 mm in diameter, the intratumoral injections were performed twice a week. Mice in the control group were intratumorally injected with PBS of the same volume. The tumor volumes were monitored every 2 days, and calculated using the following equation: 1/2 × (length × width2). The mice were sacrificed when tumor reached 1000 mm3.

## 3 Results

### 3.1 Active compounds in GLQMW

In the clinical practice of TCM, GLQMW is often used in combination with chemotherapy, or long-term application after the end of the chemotherapy cycle to treat BC. Therefore, in our study, we investigated the components of GLQMW, whether its compounds have potential anticancer effects. GLQMW is composed of five herbs: THF, FZ, FL, QM, and SY. The compounds in GLQMW that met the screening criteria were QM 1, THF 2, FL 13, FZ 16, and SY 16. A total of 48 compounds were included in our study (**Supplementary Table 1**).

### 3.2 Target of compounds prediction

**Figure 1:**
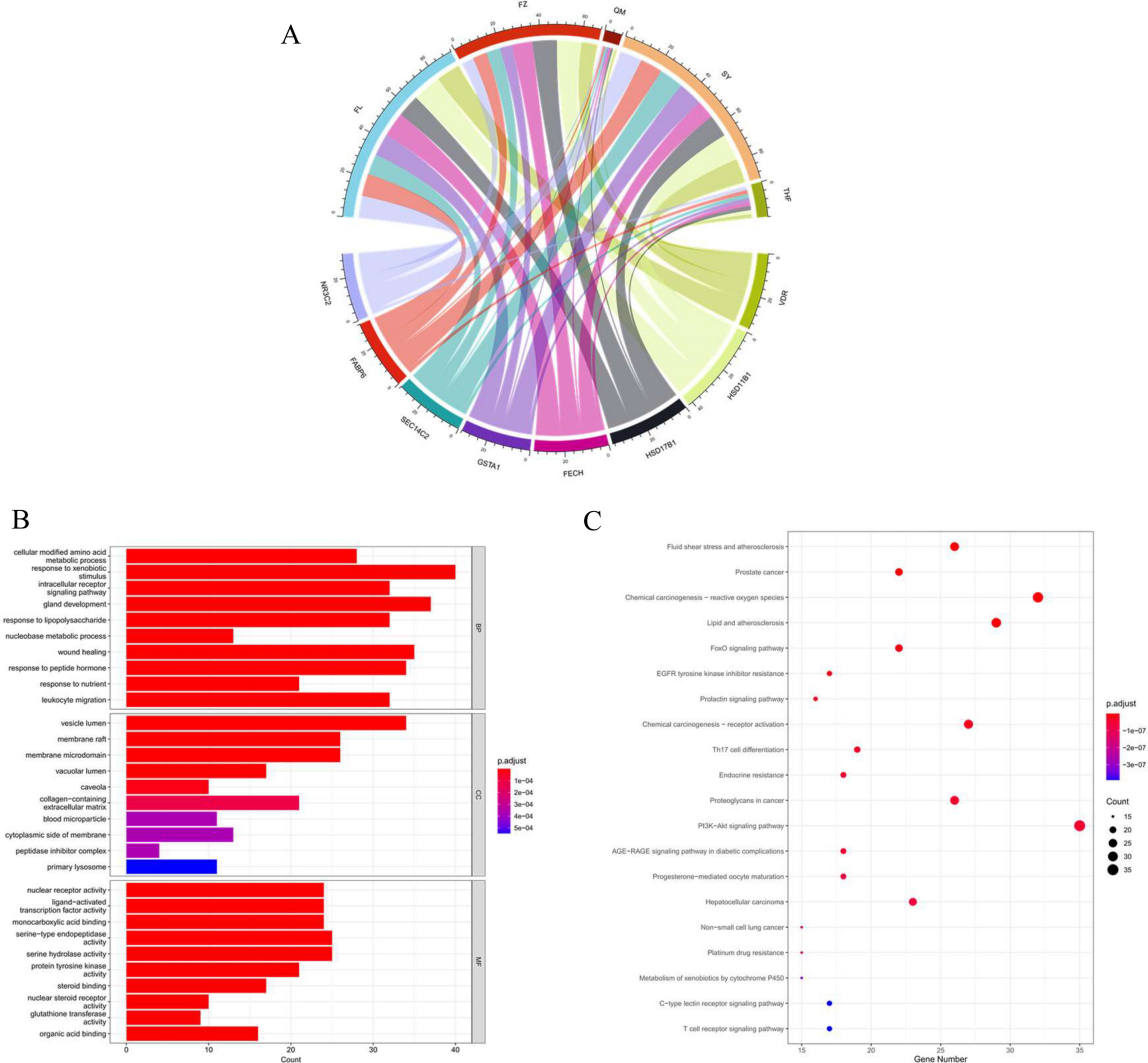
GLQMW related targets and pathways. **(A)**. Correspondence between GLQMW and main target proteins; **(B)** GO analysis of the GLQMW target proteins; **(C)** KEGG analysis of the GLQMW target proteins.

PharmMapper platform is used to input the 3D structure of compounds that meet the screening criteria in GLQMW and predict human target proteins by matching pharmacophores, which may be the basis for achieving therapeutic effects. In this study, the Z ‘score was used as the basis for determining the correlation between compounds and target proteins, and predictive targets that met the criteria (Z’ score>1.0) were included in further studies. **Figure 1A** shows the corresponding relationship between drug like compounds and predicted targets. Then, we conducted a statistical analysis of these prediction targets. Some proteins, such as corticosteroid 11-b dehydrogenase isoenzyme 1 (HSD11B1), estradiol 17-b dehydrogenase 1 (HSD17B1), vitamin D3 receptor (VDR), NR3C2, FABP6, SEC14C2, GSTA1, and FECH, may be related to the treatment of BC.

### 3.3 GO/KEGG enrichment analysis for GLQMW targets

GO enrichment analysis was performed on the target genes of GLQMW (p.adj < 0.05). The top 30 significantly enriched GO-BP terms are listed in **Figure 1B**. The results clearly demonstrated that numerous targets are involved in various biological processes associated with response to lipopolysaccharide, response to xenobiotic stimulus, leukocyte migration, which are closely related to immune reaction. The GO-CC enrichment analysis results shown in **Figure 1B** demonstrated that GLQMW acts mainly on vesicle lumen, membrane raft, and membrane microdomain. The results of GO-MF enrichment analysis shown in **Figure 1B** demonstrated that GLQMW targets are involved in nuclear receptor activity, ligand–activated transcription factor activity, monocarboxylic acid binding, bile acid binding, and glutathione transferase activity.

KEGG analysis of the GLQMW target (p < 0.05), **Figure 1C**, showed significant enrichment in immune response pathways such as T cell receptor signaling pathway, C–type lectin receptor signaling pathway, Th17 cell differentiation, and FoxO signaling pathway, which related to the observation that GLQMW can improve the immunity of patients. Pathways related to Tumor drug resistance, such as those of Platinum drug resistance, Non–small cell lung cancer, Hepatocellular carcinoma, Proteoglycans in cancer, Chemical carcinogenesis – receptor activation, EGFR tyrosine kinase inhibitor resistance, Chemical carcinogenesis – reactive oxygen species, and Prostate cancer, play an important role in cancer therapy.

### 3.4 GLQMW-related gene scores were associated with the prognosis and progress of BC

**Figure 2:**
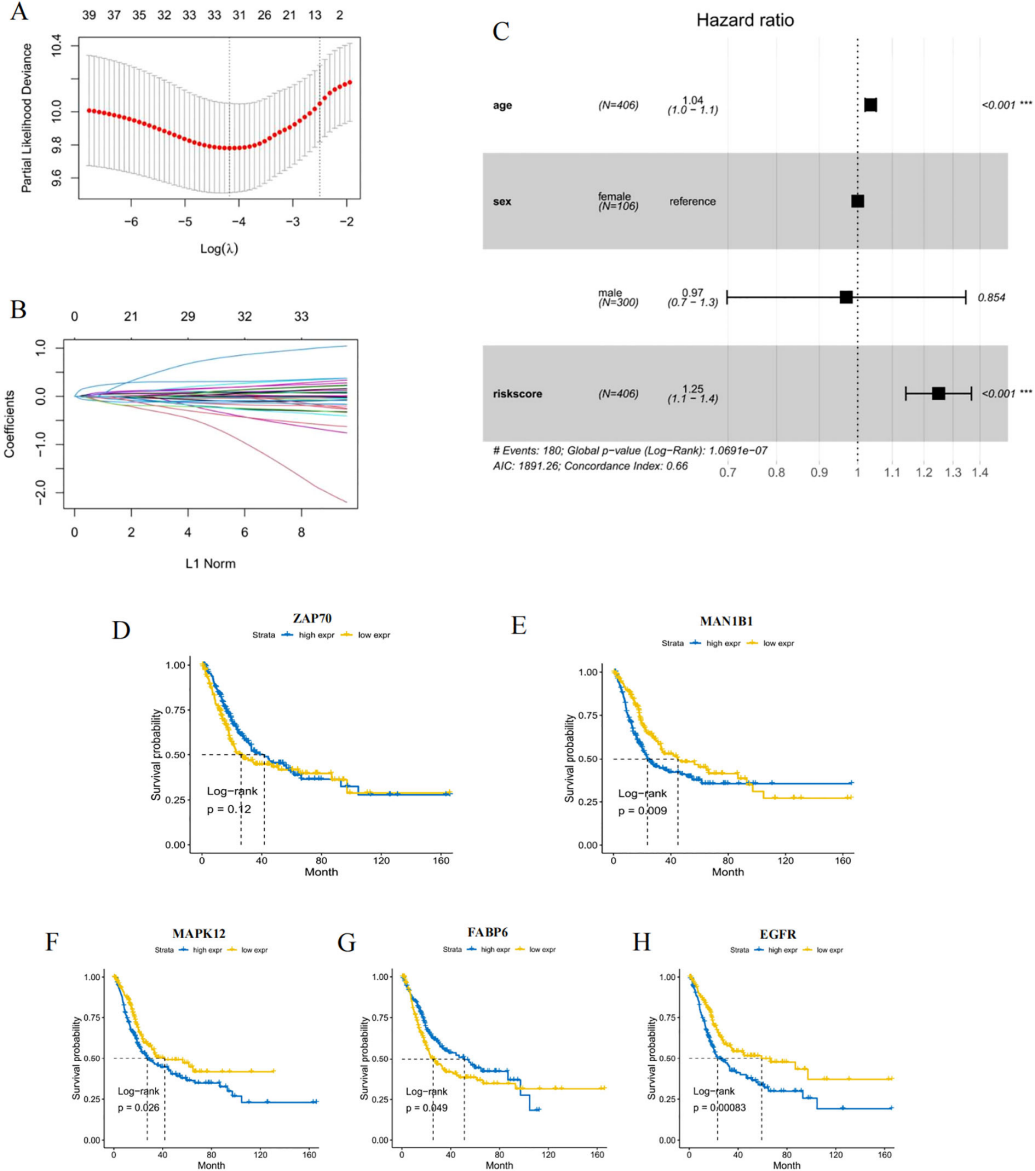
Characteristics of the prognostic gene signature. **(A)** Identification of the optimal penalization coefficient lambda in the Lasso regression model. **(B)** LASSO Cox regression algorithm was used to identify the most robust prognostic genes. **(C)** Univariate cox forest plot for age, sex and risk score. **(D–H)** Survival analysis of 5 model genes.

Although GLQMW has therapeutic effects in its application, the specific mechanism by which it exerts therapeutic effects on BC is not clear. We will explore its mechanism based on the survival risk of BC patients. Our research results indicated that the components of GLQMW can significantly affect the tumor progression and prognosis of BC, so the targeted genes related to GLQMW are used as a gene set to explore the impact of these genes on the prognosis of BC. Therefore, in order to further explore prognostic value of GLQMW-related genes, univariate cox analysis of each gene was performed to reduce the noise of genes without prognostic value (p<0.05 is considered to have prognostic value). The TCGA cohort was randomly divided into a training set and a validation set (1:1), containing randomly assigned patients with good and poor prognosis. Then, Lasso regression was used to present key genes to establish a prognostic prediction model in an internal sequence set (**Figures 2A, B**), which contained the five genes that match the model are MAPK12, MAN1B1, EGFR, FABP6, and ZAP70. The prognostic index is estimated as follows: (0.201 * MAN1B1)+(0.116 * EGFR)+(-0.124 * FABP6)+(-0.544 * ZAP70)+(0.655 * MAPK12). The model was used to calculate the risk score for each sample, and patients were divided into high-risk and low-risk groups based on the median risk score. The results of survival analysis showed that overexpression of MAPK12, MAN1B1, EGFR, and low expression of FABP6 were associated with a low overall survival rate in patients with BC (**Figures 2D–H**). Due to such significant differences in risk score groupings based on gene expression composition, we further verify whether this feature can be an independent predictor. After analyzing age, stage, and risk scores in a multivariate model, risk scores based on the five gene model remain independent prognostic factors (**Figure 2C**).

### 3.5 Development and validation of GLQMW-based prognostic model

**Figure 3:**
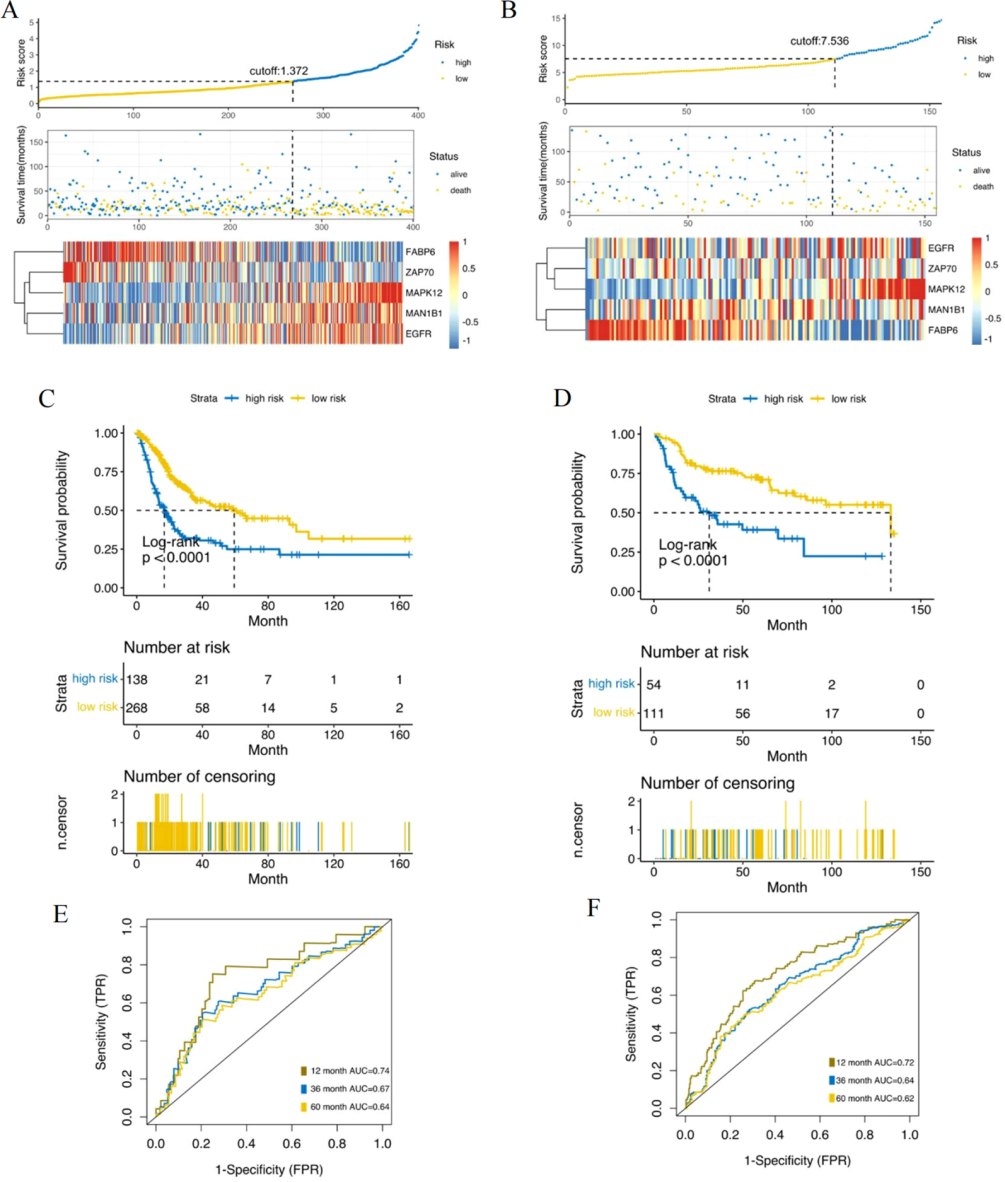
Risk score analysis, time-dependent ROC analysis and Kaplan-Meier analysis for the validation of prognostic model in TCGA set and GEO set. **(A)** Kaplan-Meier analysis in TCGA dataset; **(B)** Kaplan-Meier analysis in GEO dataset; **(C)** Time-dependent ROC analysis in TCGA dataset; **(D)** time-dependent ROC analysis in GEO dataset; **(E)** Risk score analysis and risk score group gene expression level in TCGA; **(F)** Risk score analysis and risk score group gene expression level in GEO.

The prognostic value of five gene signatures was verified in the internal validation set and the entire TCGA cohort (**Figure 3A**). In addition, we used an independent cohort (GEO dataset) to verify the prognostic value of our five genetic characteristics (**Figure 3B**). The logarithmic rank test was used to examine the overall survival difference between the two groups. To assess the effectiveness of the prognostic model, a time dependent subject performance characteristic (ROC) curve was used to assess sensitivity and specificity (**Figures 3E, F**). The results show that this feature can accurately predict the overall survival rate of BC patients in the cohort (**Figures 3C, D**). Patients with higher risk scores have significantly poorer prognosis, indicating that the five gene model also has therapeutic effects for patients, which can reverse the expression levels of the five genes to achieve therapeutic effects.

### 3.6 The differences of functional annotation and immune cell among the high- and low-risk groups and cAMP analysis

**Figure 4:**
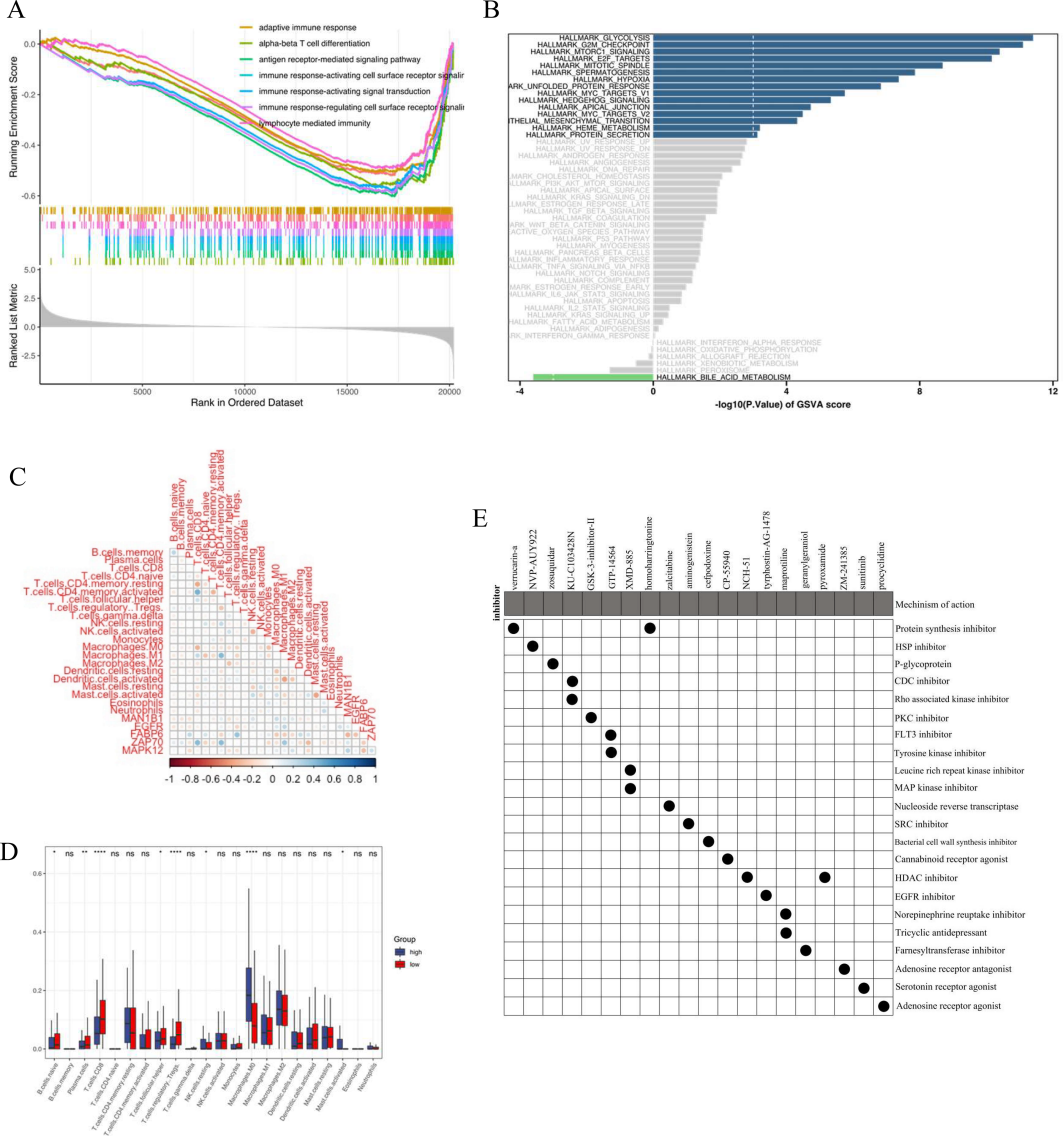
The relationship of five-gene risk groups with functional annotations and immune cell infiltration. **(A)** The Hallmark enrichment of high- and low-risk groups by GSEA method; **(B)** The Hallmark enrichment of high- and low-risk groups by GSVA method; **(C)** Correlation between model gene expression and immune cell infiltration; **(D)** Boxplot of the distribution of 22 immune cells in the high- and low-risk groups; **(E)** CMap database analysis identifies potential cancidate small molecular drugs targeting the DEGs between high- and low-groups.

In order to fully understand the relationship between risk scores and BC biology, GSEA was used to perform landmark functional enrichment based on differentially expressed genes between high-risk and low-risk groups. In the low risk group, there are adaptive immune response, alpha beta T cell differentiation, acute receptor-mediated signaling path, immune response-activating cell surface receiver signaling path, immune response-activating signal transmission, immune response-regulating cell surface receiver signaling path, and lymphocyte mediated immunity (**Figure 4A**). The GSVA results (**Figure 4B**) show that the high-risk group is enriched in glycolysis, G2M checkpoint, mTORC1 signaling, MYC targets V2, and the low-risk group is enriched in bile acid metabolism. Bile acids can regulate metabolism and inflammation through the nuclear farnesin X receptor and the takeda G protein coupled receptor 5 (34), and stable level of bile acids can play a role in inhibiting cancer (35), which is the reason why low risk BC patients have longer survival time via activating this pathway. Glycolysis is the main way for cancer cells to generate energy and cancer cells ensure sufficient energy by increasing glucose intake, while inhibiting glycolysis can make cancer cells sensitive to treatment (36). Tumor cells mainly rely on G2-M checkpoints whose destruction is a marker of cancer to stop the cell cycle and repair DNA damage (37), therefore, activation of this pathway can lead to drug resistance in tumor cells.

Then, we explored the correlation between gene expression and immune cells in the predictive model (**Figure 4C**). Among them, MAN1B1 is negatively correlated with naive B cells, plasma cells, CD8+T cells, T cells follicular helper (Tfh), and T regulatory cells (Tregs), while it is positively correlated with NK cells resting, and macrophages M0 (M0). FGFR is positively correlated with activated mast cells, M0, and negatively correlated with naive B cells, plasma cells, and Tregs. FABP6 is positively correlated with Tregs and negatively correlated with plasma cells and M0. ZAP70 is positively correlated with plasma cells and naive CD4+T cells, while negatively correlated with naive B cells, M0, and activated mast cells. MAPK12 is positively correlated with M0 and negatively correlated with plasma cells, Tfh, and Tregs.

The difference in infiltration of 22 immune cells between the high-risk group and the low-risk group was compared. The difference in infiltration of immune cells showed that compared to the high-risk group, the low-risk group could detect a higher proportion of naive B cells, plasma cells, CD8+T cells, Tfh, and Tregs. The proportion of NK cells resting, M0, and activated mast cells in the low-risk group were lower (Wilcoxon signed rank test, p<0.05) (**Figure 4D**).

According to the target of GLQMW Active ingredient, we divided it into high-risk group and low-risk group, which is also conducive to distinguish the effectiveness of GLQMW application in patients. We input the DEGs obtained into the CMap database to search for potential drugs that can be used to treat high-risk patients and reverse the ineffective treatment of GLQMW. Based on the DEGs between the high-risk and low-risk groups, the CMap mode of action (MoA) analysis showed that the inhibitors mentioned above (**Figure 4E**) and small molecule drugs with highly significant correlation share a common mechanism of action (**Supplementary Table 2**). Positive connectivity scores indicates that the drug can induce biological phenomena in human cell lines. On the contrary, negative connectivity scores indicates that the drug has reversed the required biological characteristics, thus having potential therapeutic value. Verrucarin-a, Zosuquidar, homoharringtonine, zalcitabine, aminogenistein, and cefpodoxime are potential drugs that can be used in combination with GLQMW for the treatment of BC patients.

### 3.7 Single cell analysis in BC

**Figure 5:**
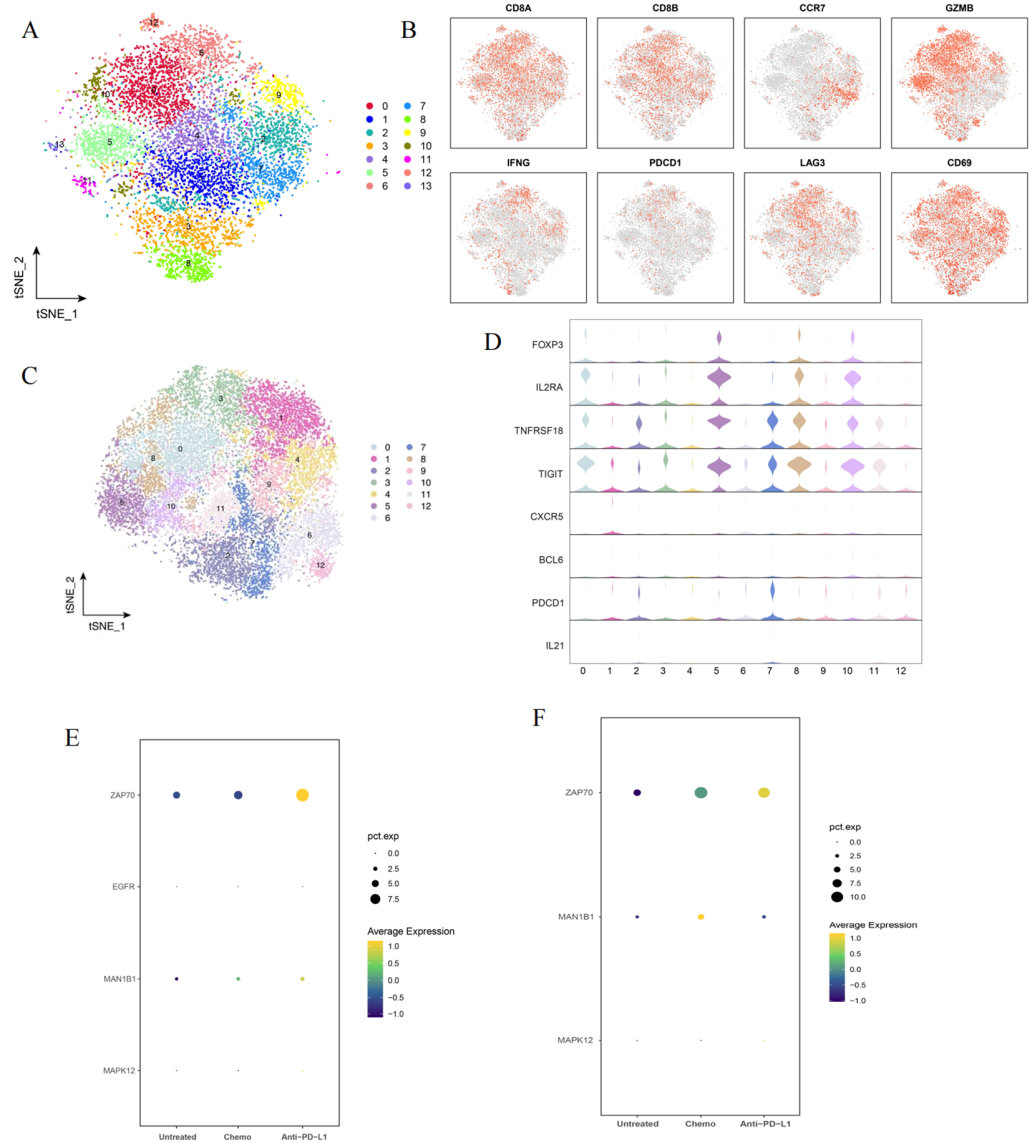
Single cell analysis of T cells in BC treatments. **(A)** Sub-cluster after CD8+ cell sorting; **(B)** Expression density map of CD8+ signature genes; **(C)** Sub-cluster after CD4+ cell sorting; **(D)** Violin plot of CD4+ signature gene expression; **(E)** Model Gene expression in CD8+ cell of different treatment groups; **(F)** Model Gene expression in CD8+ cell of different treatment groups.

We used CD8A, CD8B, CCR7, GZMB, IFNG, PDCD1, LAG3, and CD69 as marker genes dividing CD8+ cells in BC into 13 clusters (**Figures 5A, B**). CD8+ T cells are cytotoxic T cells with killing functions, which depends on CD8A and CD8B encode the α and β chains (38). CCR7 can be activated by CCL19/CCL21, and is related to the homing, activation, proliferation, and response of T cells (39). Granzyme B (GZMB) can participate in the activation of Gasdermin E (GZME) to activate pyroptosis in cancer cells (40). IFN-γ, expressed by IFNG, kills tumor cells by inducing apoptosis or non-apoptotic cell death (41). PDCD1, which encodes the immune checkpoint protein PD-1 with an ITIM domain, is expressed in activated T cells and plays an important role in the formation of peripheral immune tolerance and tumor evasion (42). LAG-3, like PD-1, functions as an immune checkpoint and is involved in the formation of immune tolerance and tumor immune evasion (43). CD69 is the earliest expressed membrane surface molecule after lymphocyte activation, playing a regulatory role, promoting tissue retention, regulating the differentiation of Th17/Treg cells, and aiding the exhaustion of resident memory T cells in the tumor microenvironment (44).

CD4+ T cells in BC, identified by such as FOXP3 and IL2RA into 12 clusters (**Figures 5C, D**). IL2RA (CD25) is the alpha chain of the interleukin 2 receptor complex, expressed on the surface of mature T cells, playing a crucial role in immune regulation (45). TNFRSF18 (GITR) is a member of the tumor necrosis factor family, involved in the proliferation of Treg cells and the activation of CD4 Th cells (46). IL-21 is a gamma chain cytokine family member primarily produced by CD4+ T cells and natural killer T (NKT) cells, exhibiting certain anti-tumor effects (47).

Then, we analyzed the expression levels of ZAP70, EGFR, MAN1B1 and MAPK12 in CD8 and Treg cells in different treatment groups (**Figures 5E, F**). ZAP70 and MAN1B1 in the chemotherapy and immunotherapy groups, the expression of MAN1B1 increased, while ZAP70 did not change significantly in the chemotherapy group, but increased in the immunotherapy group. For Treg cells, the expression of ZAP70 increased after treatment, but MAN1B1 expression increased only in the chemotherapy group. The increased expression of ZAP70 and MAN1B1 in the chemotherapy and immunotherapy groups may lead to the activation of immune cells and the reduction of immunosuppression.

### 3.8 Drug similarity analysis and macromolecular docking

**Figure 6:**
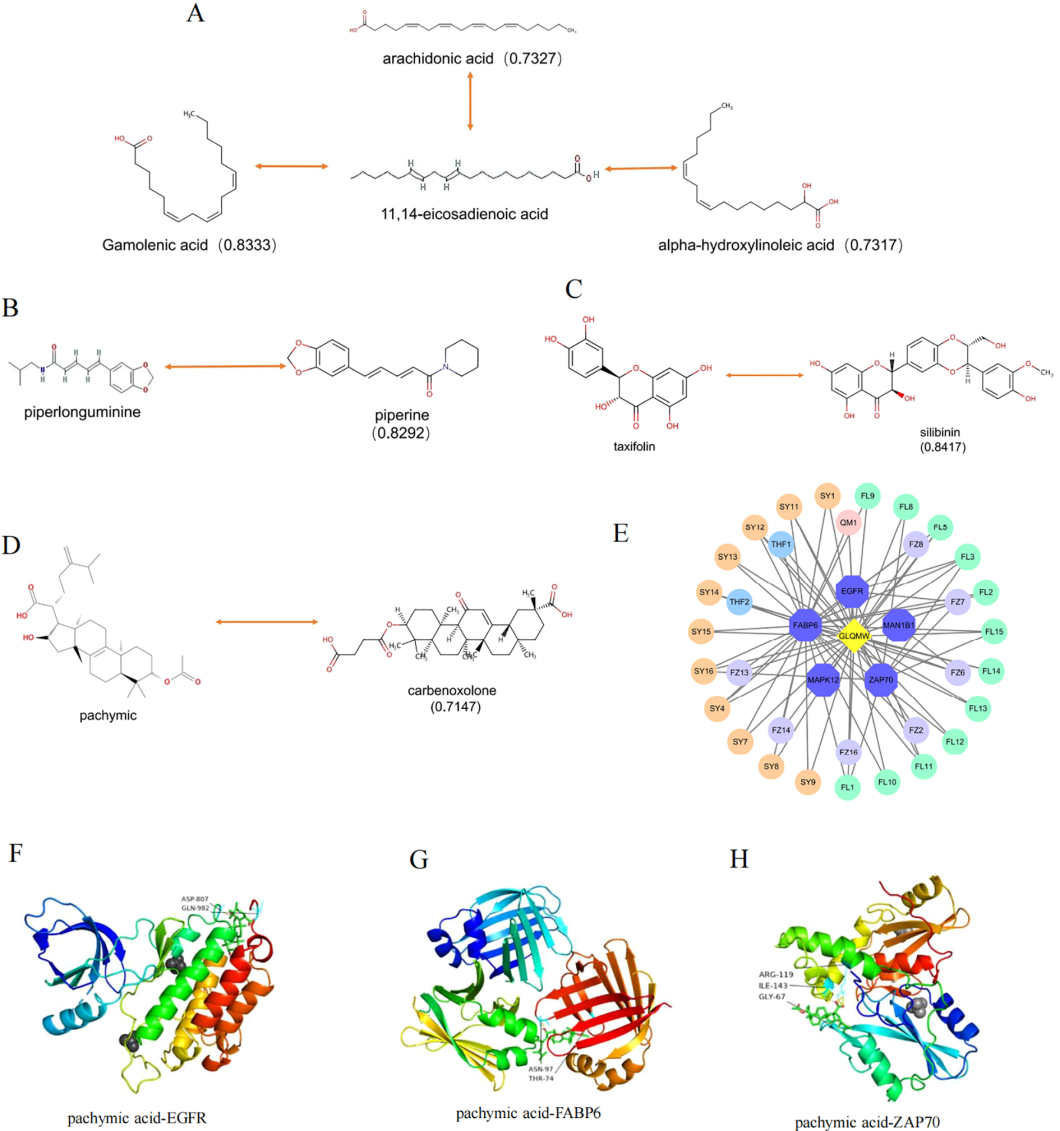
Drug similarity between compounds and drugs of DrugBank, docking mode and interactions between compounds and target proteins. **(A–D)** Drug similarity of compounds; **(E)** Relationship between model target and compound; **(F–H)** Molecular docking model of target and compound. The two-way line represents the comparative relationship between the compound and the drug, and the numbers represent the drug similarity.

Based on the RDKit tool, the small-molecule compounds obtained were compared with all small-molecule drugs released from the DrugBank database, using 0.6 as the baseline to export similar drugs. The drug similarities of 11,14-eicosadienoic acid, gamolenic acid, arachidonic acid, and alpha-hydroxylinoleic acid were 0.8333, 0.7327, and 0.7317, respectively (**Figure 6A**).

Piperlonguminine has similarity scores of 0.8292 with piperine and 0.6122 with isoeugenol (**Figure 6B**). Additionally, the similarity scores between pachymic acid and carbenoxolone, as well as between taxifolin and silibinin, are 0.7147 and 0.8417, respectively (**Figures 6C, D**). **Figure 6E** shows the relationship between model target and active compounds.

Pachymic acid bound to EGFR, forming hydrogen bond interactions with residues Asp-807 and Gln-982; FABP6, forming hydrogen bond interactions with residues Asn-97 and Thr-74; ZAP70, forming hydrogen bond interactions with residues Arg-119, Gly-67 and Ile-143 (**Figures 6F–H**).

### 3.9 Pan-cancer analysis

TIMER2 is used to evaluate the differential expression of MAPK12, MAN1B1, EGFR, FABP6, and ZAP70 between tumors and adjacent normal tissues in the TCGA database. As shown in **Supplementary Figure 1**, MAN1B1 expression is higher in urinary system cancers, such as bladder urothelial carcinoma (BLCA), kidney renal clear cell carcinoma (KIRC), and prostate cancer (PRAD) (p<0.001) than in normal tissue. FABP6 expression shows a significant decrease in BLCA (p<0.01) and KIRC (p<0.001). MAPK12, EGFR, and ZAP70 also exhibit significant differential expression in KIRC (p<0.001) and PRAD (MAPK12, EGFR: p<0.001; ZAP70: p<0.01). Additionally, these genes show significant differential expression in other tumors, such as kidney papillary cell carcinoma (KIRP) and head and neck squamous cell carcinoma (HNSC).

To explore the association between the expression of MAPK12, MAN1B1, EGFR, FABP6, and ZAP70 and infiltrating immune cells, we used TIMER to analyze their expression in BLCA, KIRC, and PRAD alongside various immune cells, including B cells, CD8+ T cells, CD4+ T cells, macrophages, neutrophils, and dendritic cells. As shown in **Supplementary Figure 2** and **Supplementary Table 3**, in BLCA, MAPK12, MAN1B1, EGFR, FABP6, and ZAP70 all exhibit strong correlations with immune cell infiltration. In PRAD and KIRC, EGFR and ZAP70 show correlations with multiple immune cell infiltrations, while MAPK12, MAN1B1, and FABP6 are associated with some immune cells.

### 3.10 Inhibitory effect of pachymic acid on T24 and MB49 cells

**Figure 7:**
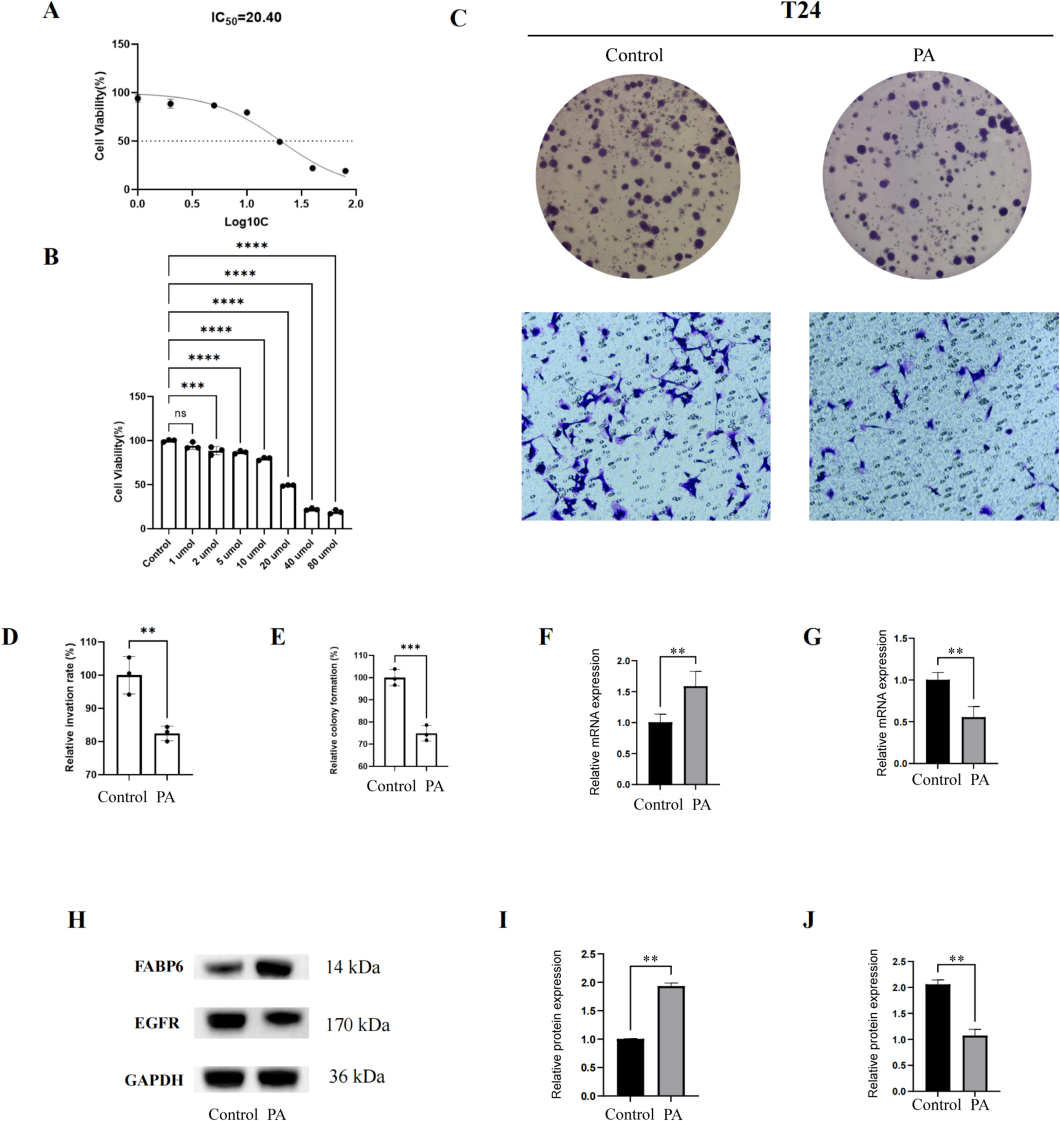
Inhibition of pachymic acid on the activity of T24 cells. **(A)** IC50 curve of pachymic acid in T24 cells; **(B)** Killing effect of different concentrations of polycyclic acid on T24 cells; **(C)** Effect of pachymic acid on proliferation and invasion of T24 cells; **(D)** Differences in the proportion of invasion after drug treatment; **(E)** Differences in cell clonal proliferation after drug treatment; **(F)** Real-Time PCR (qPCR) to assess the expression levels of FABP6 in T24 cells, the horizontal axis represents different treatment groups, and the vertical axis represents gene expression levels; **(G)** Real-Time PCR (qPCR) to assess the expression levels of EGFR in T24 cells; **(H)** Western blot showed the protein levels of FABP6, EGFR and GAPDH in different treatment T24 cells; The bar chart of I and J shows ratio differences in the protein level of FABP6/GAPDH, and EGFR/GAPDH. *, p<0.05; **, p<0.01; ***, p<0.001; ****, p<0.0001.

**Figure 8:**
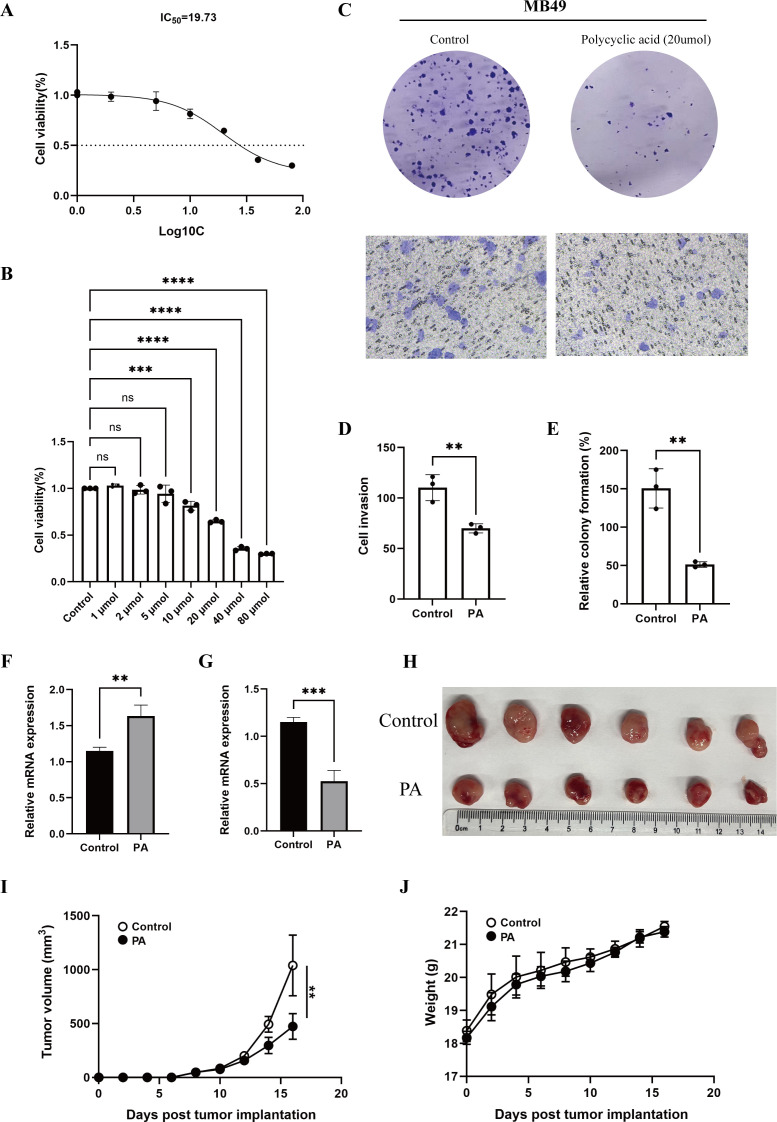
Inhibition of pachymic acid on the activity of MB49 cells. **(A)** IC50 curve of pachymic acid in MB49 cells; **(B)** Killing effect of different concentrations of pachymic acid on MB49 cells; **(C)** Effect of pachymic acid on proliferation and invasion of MB49 cells; **(D)** Differences in the proportion of invasion after drug treatment; **(E)** Differences in cell clonal proliferation after drug treatment; **(F)** Real-Time PCR (qPCR) to assess the expression levels of FABP6 in MB49 cells, the horizontal axis represents different treatment groups, and the vertical axis represents gene expression levels; **(G)** Real-Time PCR (qPCR) to assess the expression levels of EGFR in MB49 cells; **(H)** *In vivo* experiment, tumors were dissected and photographed. **(I, J)** The size of the tumors and the weight of mice were monitored regularly (n=6). *, p<0.05; **, p<0.01; ***, p<0.001; ****, p<0.0001.

When different concentrations were used to determine the killing effect of PA on T24 and MB49 cells, 20um was the concentration of PA that could cause effective killing after treatment, and showed half killing near this concentration (**Figures 7A, B**, **8A, B**). **Figures 7C–E** and **Figures 8C–E** shows that after 20um PA treatment, the cell proliferation ability was significantly reduced, and the invasion ability was also decreased compared with the untreated group. After drug treatment, the expression level of FABP6 was significantly up-regulated than that of the control group (**Figure 7F**, **8F**), while the expression level of EGFR was significantly down regulated (**Figure 7G**, **8G**), which indicated that PA could regulate the level of lipid metabolism and inhibit the basic mechanism of tumor progression. The upregulation of FABP6 and inhibition of EGFR were also demonstrated at the protein level in T24 cells (**Figures 7H–J**). In addition, *in vivo* experiments demonstrated that intratumoral administration of PA significantly suppressed bladder cancer progression (**Figures 8H, I**). PA treatment was well tolerated in mice, with no apparent signs of drug-induced toxicity (**Figure 8J**).

## 4 Discussion

Bladder cancer is a serious disease of urinary system, belonging to the category of “Longbi”, and characterized by the accumulation of heat and toxin in the body, which lead to the occurrence of BC in the long pathological progression. Chinese medicine experts believe that the treatment of BC should be based on the principle of clearing away heat and detoxification, tonifying the kidney and resolving phlegm. GLQMW has QM, FL, FZ, THF and SY, which has the functions of clearing heat and diuresis, resolving phlegm and removing stasis, and tonifying the spleen and kidney, so it is suitable for the treatment of BC. In the long-term clinical application of TCM, TCM doctors have summarized that GLQMW has curative effect on patients with BC, which embodied in that GLQMW can effectively improve the quality of life and survival time of patients. Our research is based on clinical experience and combined with bioinformatics principles to analyze the mechanism of GLQMW on BC.

Based on TCGA and GEO datasets, we established a risk prediction model for bladder cancer, and linked the expression level of genes that can affect the survival of bladder cancer patients with the prognosis of bladder cancer. The gene expression profile of patients with good prognosis meets the low expression of MAPK12, MAN1B1, EGFR and the high expression of FABP6 and ZAP70. Fatty acid binding protein 6 (FABP6), a metabolic related gene, has been shown to be a gene with high expression levels that is beneficial to patient survival (48), and can regulate the proportion of CD8+T cells in the immune microenvironment, which can be consistent with the improvement of immune infiltration in the metabolic microenvironment. As a biomarker for evaluating the prognosis and degree of immune infiltration in patients with BC (49), Wang et al. found that silencing MAN1B1 can inhibit AKT signaling pathway activity to hinder cell proliferation and promote apoptosis in BC cell (50), which is consistent with our results. ZAP70, protein tyrosine kinase (PTK) substrate protein, can activate T cell antigen receptors (TCRs) and their downstream pathways (51), which can regulate T cell activity and quantity (52). In our results, we found that the expression of ZAP70 is downregulated in patients with low survival rates, indicating low immune activity in patients, and GLQMW targets this gene to reconstruct the patient’s immune environment. MAPK12 (53) and EGFR (54), star oncogenes, are often used as targets for anticancer drugs to develop iterative drugs, which are included in the treatment targets of models and GLQMW. The five model genes can have an impact on the proportion of immune cells in the immune microenvironment, mainly including naive B cells, plasma cells, CD8+T cells, T cells follicular helper (Tfh), T regulatory cells (Tregs), NK cells resting, resting NK cells, macrophages M0, and activated mast cells, which are believed to be new targets for immune microenvironment regulation due to affecting both survival and immune cell ratio. Medical workers can use five gene prognosis models to classify the survival status of patients and take corresponding measures to prevent patients from having worse outcomes. Through network pharmacology, we discovered compounds in GLQMW, including 6-methylhept-5-enoic acid, piperlonguminine, Methylcimicifugoside_qt, taxifolin, and pachymic acid, which exhibit potential therapeutic effects on bladder cancer (BC). After conducting drug similarity analysis, we found that drug molecules similar to these include Gamolenic acid, alpha-hydroxylinoleic acid, oleandrin, glycyrrhizic acid, peoniflorin, hydrocortisone, prednisolone, rimexolone, methylprednisolone, melengestrol, vamorolone, carbenoxolone, piperine, isoeugenol, Vamorolone, and silibinin. Among them, silibinin, Gamolenic acid, piperine, alpha-hydroxylinoleic acid, carbenoxolone, peoniflorin, vamorolone, and isoeugenol exhibit strong antitumor effects.

Silibinin upregulates death receptor 5 after activating AMPK to promote the death of liver cancer cells (55). Gamolenic acid activates the ASK1-p38 MAPK pathway by increasing ROS concentrations, inducing apoptosis in cancer sarcoma cells (56). Piperine binds to estrogen receptor-a, altering the expression of the DNA-PK complex and DDR proteins, thereby enhancing the sensitivity of breast cancer cells to radiation (57). Alpha-hydroxylinoleic acid inhibits the PI3K/AKT/mTOR signaling axis by upregulating TRIB3 expression, causing cell death in endometrial cancer (EC) cells without affecting normal endometrial cells (58). Carbenoxolone reduces neuroblastoma proliferation by inhibiting PANX1 (59). Peoniflorin may inhibit STAT3/PD-L1 signaling by enhancing SOCS3, thereby restoring T-cell sensitivity to kill liver cancer cells (60). Vamorolone exhibits anti-inflammatory effects by reducing inflammatory cytokines in the NF-κB pathway in primary DIPG cells from mice, suggesting potential antitumor effects (61). Isoeugenol inhibits HT29 cell proliferation and induces apoptosis by increasing the Bax/Bcl2 ratio and mRNA expression of Caspase-9 and Caspase-3 (62).

Therefore, the main targets of GLQMW are AMPK, ASK1-p38 MAPK, estrogen receptor-α, TRIB3, SOCS3, and Bax/Bcl2, which are primarily related to cell apoptosis or growth inhibition. This discovery may be the basis for the therapeutic effect of GLQMW. According to the GO/KEGG results for GLQMW targets, GLQMW has been shown to be related to cell proliferation-related pathways such as the MAPK, PI3K-AKT, NF-kB and STAT3/PD-L1 signaling pathways. Based on the aforementioned analysis, GLQMW inhibits cell growth or induces or promotes cell apoptosis by activating or inhibiting different signaling pathways to achieve the purpose of tumor treatment.

## 5 Conclusion

GLQMW prolongs the survival time of patients with BC by targeting prognostic related genes, including MAPK12, MAN1B1, EGFR, FABP6 and ZAP60.

## Data Availability

The datasets presented in this study can be found in online repositories. The names of the repository/repositories and accession number(s) can be found in the article/**Supplementary Material**.
