# Supplementary material for: An exploration of GuaLouQuMaiWan related novel prognostic model and search for potential compound pachymic acid in bladder cancer
Source: Front Oncol. 2026 Apr 15;16:1714939. doi: 10.3389/fonc.2026.1714939 (PMC13124571; doi:10.3389/fonc.2026.1714939)
Supplement: Supplementary file 1 [file DataSheet1.docx]

Supplementary Table 1 Properties of Effective Drug-like Compounds of GLQMW

| number | compound | structure | OB（%） | Caco-2 | DL | herb |
| --- | --- | --- | --- | --- | --- | --- |
| QM1 | dianoside A_qt | 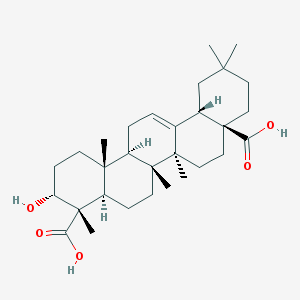 | 37.17 | 0.01 | 0.73 | Dianthi Herba |
| THF1 | Schottenol | 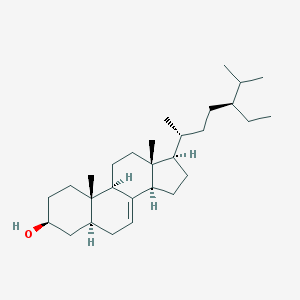 | 37.42 | 1.33 | 0.75 | Trichosanthis Radix |
| THF2 | Spinasterol | 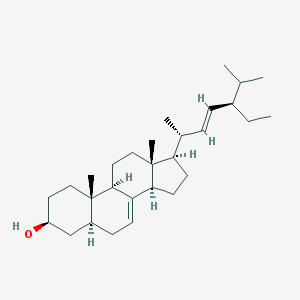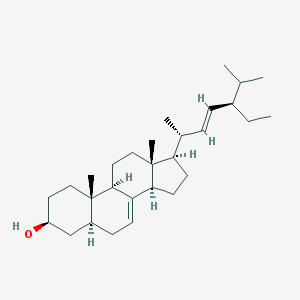 | 42.98 | 1.44 | 0.76 | Trichosanthis Radix |
| FL1 | (2R)-2-[(3S,5R,10S,13R,14R,16R,17R)-3,16-dihydroxy-4,4,10,13,14-pentamethyl-2,3,5,6,12,15,16,17-octahydro-1H-cyclopenta[a]phenanthren-17-yl]-5-isopropyl-hex-5-enoic acid | 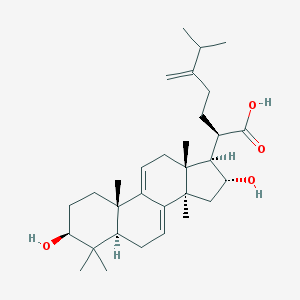 | 31.07 | 0.05 | 0.82 | Poria Cocos (Schw.) Wolf. |
| FL2 | hederagenin | 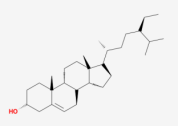 | 36.91 | 1.32 | 0.75 | Poria Cocos (Schw.) Wolf. |
| FL3 | pachymic acid | 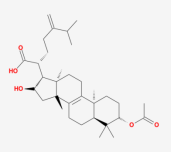 | 33.63 | 0.1 | 0.81 | Poria Cocos (Schw.) Wolf. |
| FL4 | poricoic acid C | 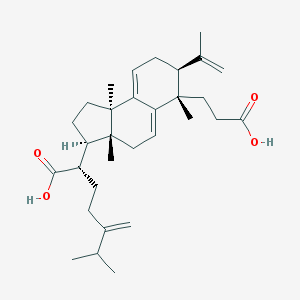 | 38.15 | 0.32 | 0.75 | Poria Cocos (Schw.) Wolf. |
| FL5 | trametenolic acid | 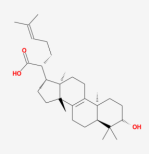 | 38.71 | 0.52 | 0.8 | Poria Cocos (Schw.) Wolf. |
| FL6 | (2R)-2-[(3S,5R,10S,13R,14R,16R,17R)-3,16-dihydroxy-4,4,10,13,14-pentamethyl-2,3,5,6,12,15,16,17-octahydro-1H-cyclopenta[a]phenanthren-17-yl]-6-methylhept-5-enoic acid | 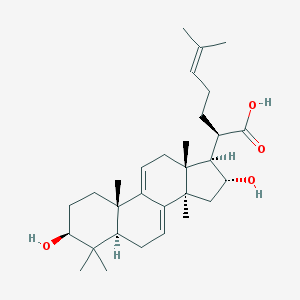 | 30.93 | 0.01 | 0.81 | Poria Cocos (Schw.) Wolf. |
| FL7 | (2R)-2-[(5R,10S,13R,14R,16R,17R)-16-hydroxy-3-keto-4,4,10,13,14-pentamethyl-1,2,5,6,12,15,16,17-octahydrocyclopenta[a]phenanthren-17-yl]-5-isopropyl-hex-5-enoic acid | 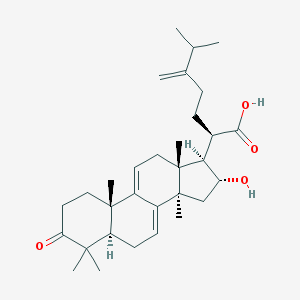 | 38.26 | 0.12 | 0.82 | Poria Cocos (Schw.) Wolf. |
| FL8 | 3beta-Hydroxy-24-methylene-8-lanostene-21-oic acid | 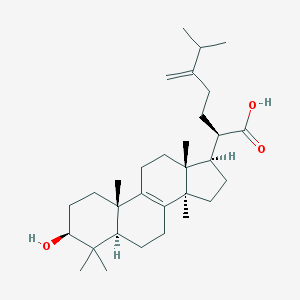 | 38.7 | 0.61 | 0.81 | Poria Cocos (Schw.) Wolf. |
| FL9 | 7,9(11)-dehydropachymic acid | 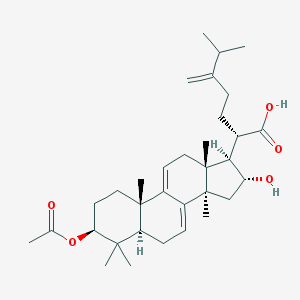 | 35.11 | 0.03 | 0.81 | Poria Cocos (Schw.) Wolf. |
| FL10 | Cerevisterol | 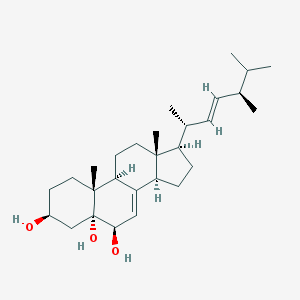 | 37.96 | 0.28 | 0.77 | Poria Cocos (Schw.) Wolf. |
| FL11 | dehydroeburicoic acid | 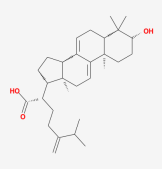 | 44.17 | 0.38 | 0.83 | Poria Cocos (Schw.) Wolf. |
| FL12 | ergosta-7,22E-dien-3beta-ol | 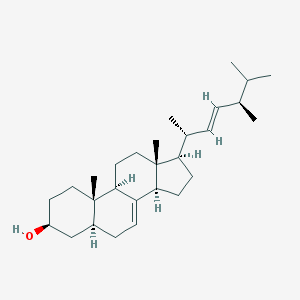 | 43.51 | 1.32 | 0.72 | Poria Cocos (Schw.) Wolf. |
| FL13 | Ergosterol peroxide | 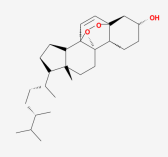 | 40.36 | 0.84 | 0.81 | Poria Cocos (Schw.) Wolf. |
| FZ1 | Neokadsuranic acid B | 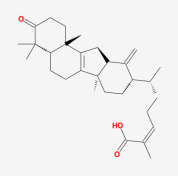 | 43.1 | 0.69 | 0.85 | Aconiti Lateralis Radix Praeparata |
| FZ2 | Carnosifloside I_qt | 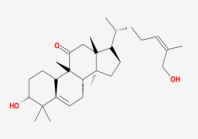 | 38.16 | 0.28 | 0.8 | Aconiti Lateralis Radix Praeparata |
| FZ3 | sitosterol | 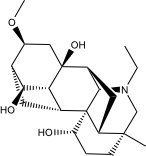 | 36.91 | 1.32 | 0.75 | Aconiti Lateralis Radix Praeparata |
| FZ4 | karakoline | 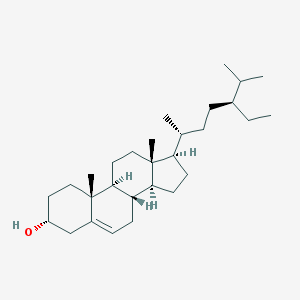 | 51.73 | 0.32 | 0.73 | Aconiti Lateralis Radix Praeparata |
| FZ5 | isotalatizidine | 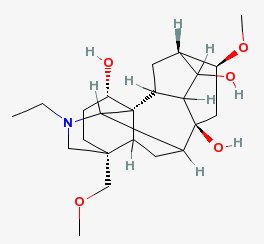 | 50.82 | -0.11 | 0.73 | Aconiti Lateralis Radix Praeparata |
| FZ6 | 6-Demethyldesoline | 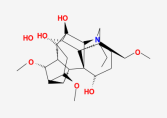 | 51.87 | -0.26 | 0.66 | Aconiti Lateralis Radix Praeparata |
| FZ7 | benzoylnapelline | 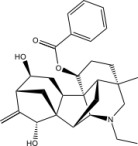 | 34.06 | 0.19 | 0.53 | Aconiti Lateralis Radix Praeparata |
| FZ8 | Deltoin | 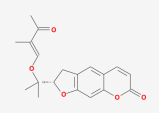 | 46.69 | 0.55 | 0.37 | Aconiti Lateralis Radix Praeparata |
| FZ9 | Karanjin | 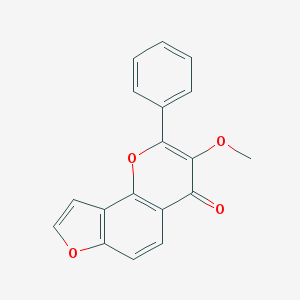 | 69.56 | 1.22 | 0.34 | Aconiti Lateralis Radix Praeparata |
| FZ10 | Deoxyandrographolide | 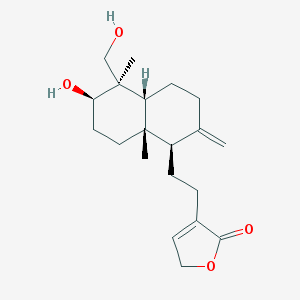 | 56.3 | 0.18 | 0.31 | Aconiti Lateralis Radix Praeparata |
| FZ11 | Delphin_qt | 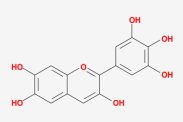 | 57.76 | 0.12 | 0.28 | Aconiti Lateralis Radix Praeparata |
| FZ12 | hypaconitine | 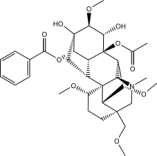 | 31.39 | -0.34 | 0.26 | Aconiti Lateralis Radix Praeparata |
| FZ13 | ignavine | 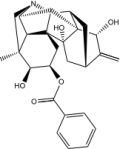 | 84.08 | -0.07 | 0.25 | Aconiti Lateralis Radix Praeparata |
| FZ14 | deoxyaconitine | 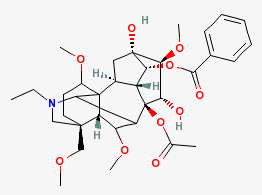 | 30.96 | -0.23 | 0.24 | Aconiti Lateralis Radix Praeparata |
| FZ15 | (R)-Norcoclaurine | 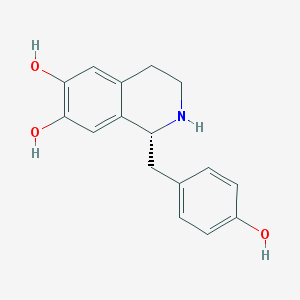 | 82.54 | 0.63 | 0.21 | Aconiti Lateralis Radix Praeparata |
| FZ16 | 11,14-eicosadienoic acid | 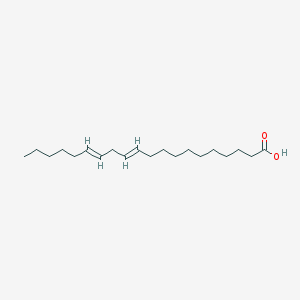 | 39.99 | 1.22 | 0.2 | Aconiti Lateralis Radix Praeparata |
| SY1 | piperlonguminine | 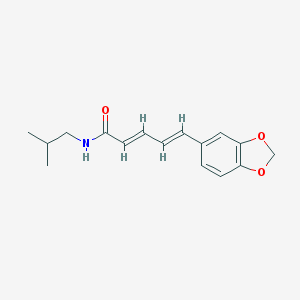 | 30.71 | 0.95 | 0.18 | Rhizoma Dioscoreae |
| SY2 | Methylcimicifugoside_qt | 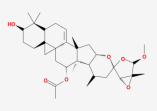 | 31.69 | 0.21 | 0.24 | Rhizoma Dioscoreae |
| SY3 | (-)-taxifolin | 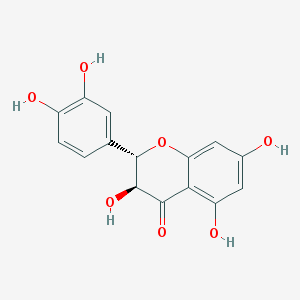 | 60.51 | -0.24 | 0.27 | Rhizoma Dioscoreae |
| SY4 | hancinol | 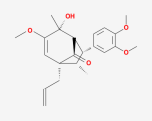 | 64.01 | 0.53 | 0.37 | Rhizoma Dioscoreae |
| SY5 | Kadsurenone | 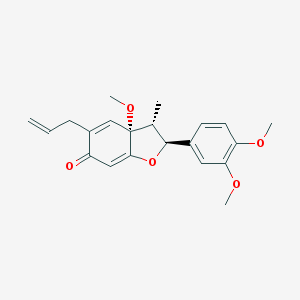 | 54.72 | 0.82 | 0.38 | Rhizoma Dioscoreae |
| SY6 | Denudatin B | 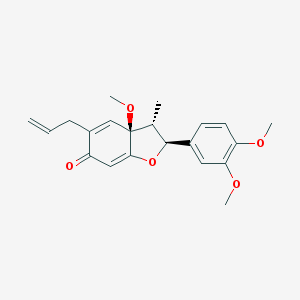 | 61.47 | 0.9 | 0.38 | Rhizoma Dioscoreae |
| SY7 | hancinone C | 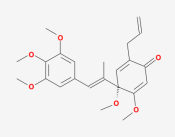 | 59.05 | 0.74 | 0.39 | Rhizoma Dioscoreae |
| SY8 | Doradexanthin | 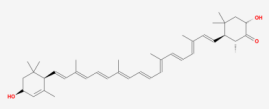 | 38.16 | 0.52 | 0.54 | Rhizoma Dioscoreae |
| SY9 | CLR | 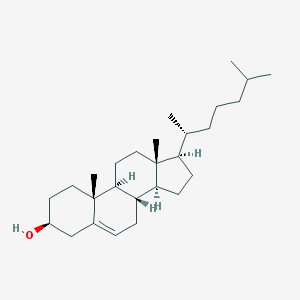 | 37.87 | 1.43 | 0.68 | Rhizoma Dioscoreae |
| SY10 | campesterol | 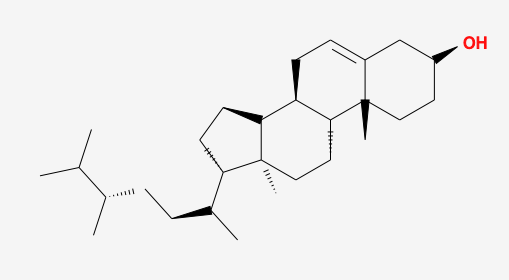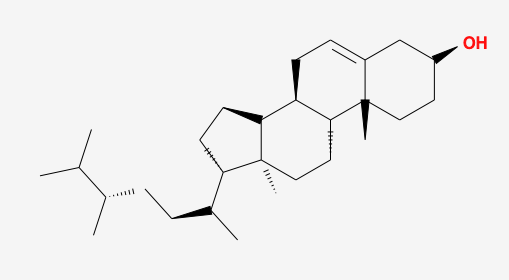 | 37.58 | 1.34 | 0.71 | Rhizoma Dioscoreae |
| SY11 | 24-Methylcholest-5-enyl-3belta-O-glucopyranoside_qt | 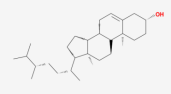 | 37.58 | 1.33 | 0.72 | Rhizoma Dioscoreae |
| SY12 | Stigmasterol | 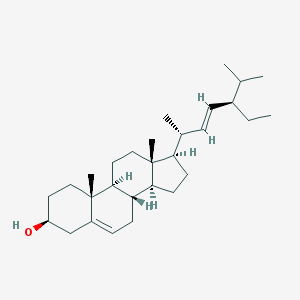 | 43.83 | 1.44 | 0.76 | Rhizoma Dioscoreae |
| SY13 | Isofucosterol | 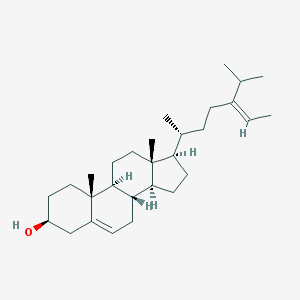 | 43.78 | 1.36 | 0.76 | Rhizoma Dioscoreae |
| SY14 | AIDS180907 | 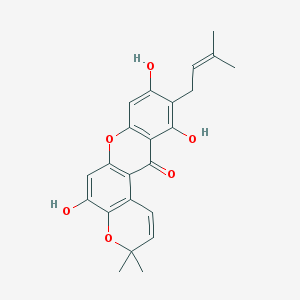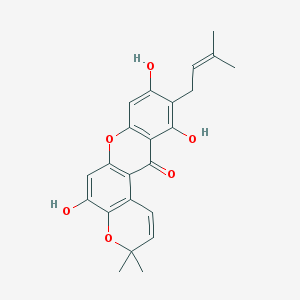 | 45.33 | 0.73 | 0.77 | Rhizoma Dioscoreae |
| SY15 | diosgenin | 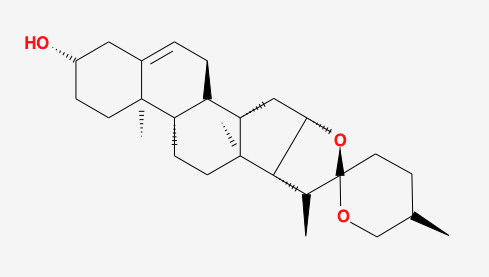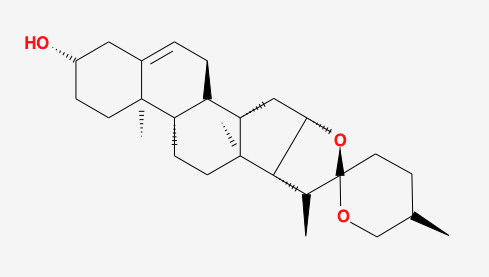 | 80.88 | 0.82 | 0.81 | Rhizoma Dioscoreae |
| SY16 | Dioscoreside C_qt | 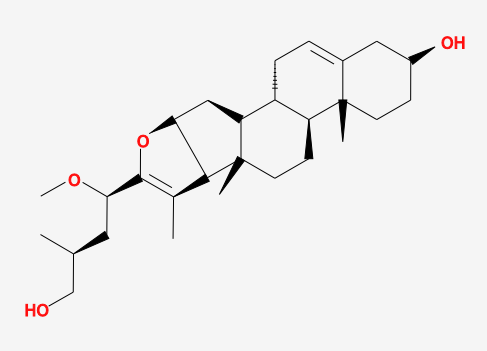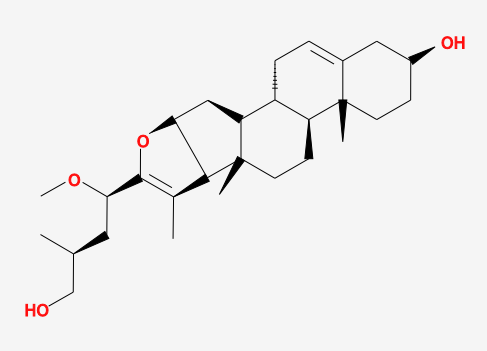 | 36.38 | 0.39 | 0.87 | Rhizoma Dioscoreae |

Supplementary Table 2.Results of CMap analysis (score <−65).

| Score | Name | Target | MOA |
| --- | --- | --- | --- |
| -89.18 | verrucarin-a |  | Protein synthesis inhibitor |
| -87.1 | NVP-AUY922 | HSP90AA1, HSP90AA2, HSP90AB1 | HSP inhibitor |
| -82.71 | zosuquidar | ABCB1, ABCB4 | P-glycoprotein inhibitor |
| -81.65 | KU-C103428N | NFE2 | CDC inhibitor, Rho associated kinase inhibitor |
| -75.46 | GSK-3-inhibitor-II | GSK3B | PKC inhibitor |
| -75.45 | GTP-14564 | FLT3, CSF1R, KIT, PDGFRB | FLT3 inhibitor, Tyrosine kinase inhibitor |
| -74.21 | XMD-885 | LRRK2, MAPK7 | Leucine rich repeat kinase inhibitor, MAP kinase inhibitor |
| -72.86 | homoharringtonine | RPL3 | Protein synthesis inhibitor |
| -71.28 | zalcitabine |  | Nucleoside reverse transcriptase inhibitor |
| -70.49 | aminogenistein | LCK | SRC inhibitor |
| -69.45 | cefpodoxime |  | Bacterial cell wall synthesis inhibitor |
| -69.08 | CP-55940 | CNR1, CNR2, CXCR4, GPR55 | Cannabinoid receptor agonist |
| -68.07 | NCH-51 | HDAC1, HDAC10, HDAC11, HDAC2, HDAC3, HDAC4, HDAC5, HDAC6, HDAC7, HDAC8, HDAC9 | HDAC inhibitor |
| -67.05 | tyrphostin-AG-1478 | EGFR, MAPK14 | EGFR inhibitor |
| -66.8 | maprotiline | SLC6A2, ADRA1A, ADRA1B, ADRA1D, ADRA2A, ADRA2B, ADRA2C, CHRM1, CHRM2, CHRM3, CHRM4, CHRM5, DRD2, HRH1, HTR2A, HTR2C, HTR7 | Norepinephrine reuptake inhibitor, Tricyclic antidepressant |
| -66.58 | geranylgeraniol |  | Farnesyltransferase inhibitor |
| -66.41 | pyroxamide | HDAC1 | HDAC inhibitor |
| -66.3 | ZM-241385 | ADORA2A, ADORA2B, ADORA1, ADORA3 | Adenosine receptor antagonist |

Supplementary Table 3. The relationship between immune cells and gene expression in urinary tumors

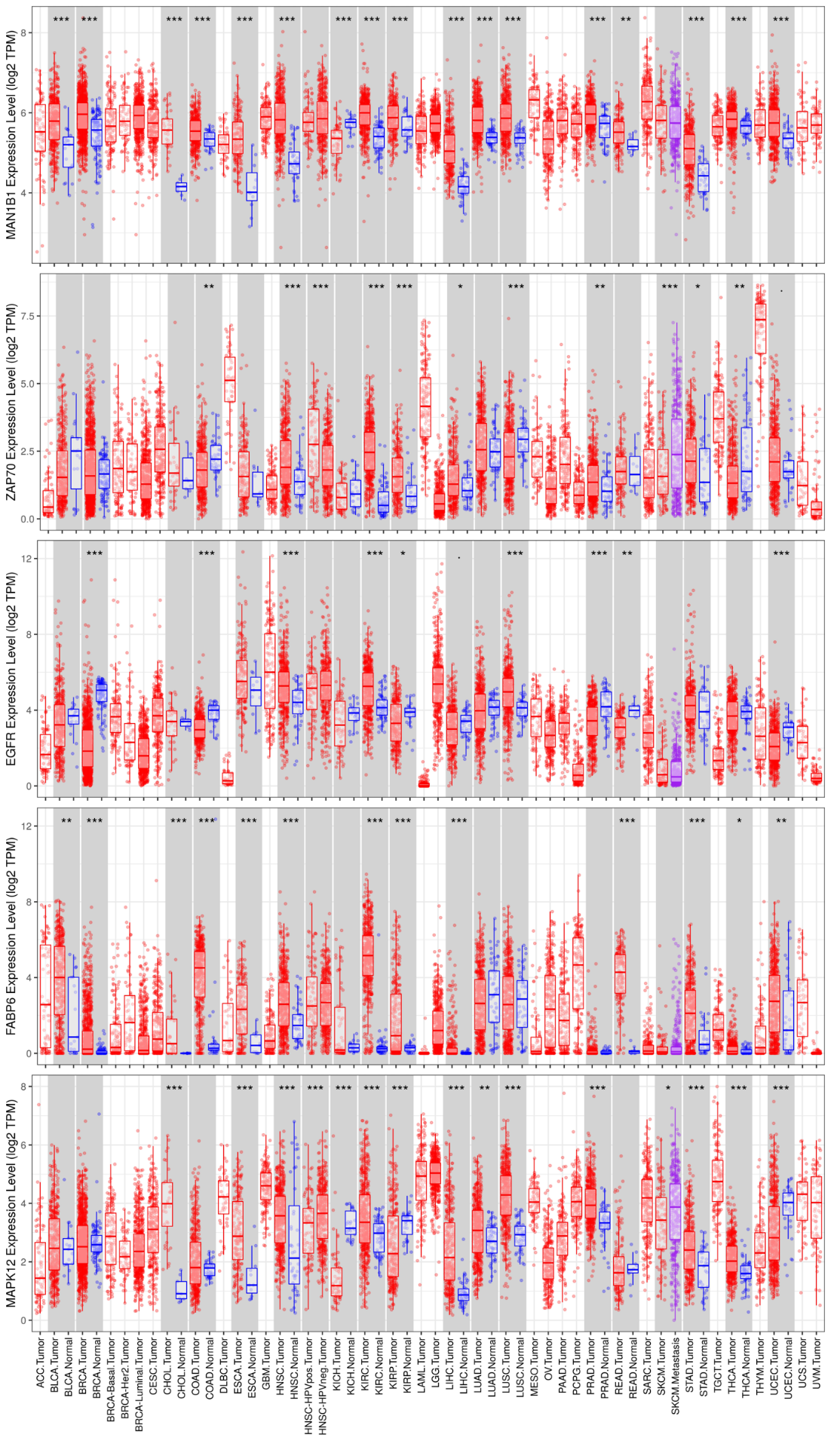


Supplementary Figure 1. Pan cancer analysis of model genes


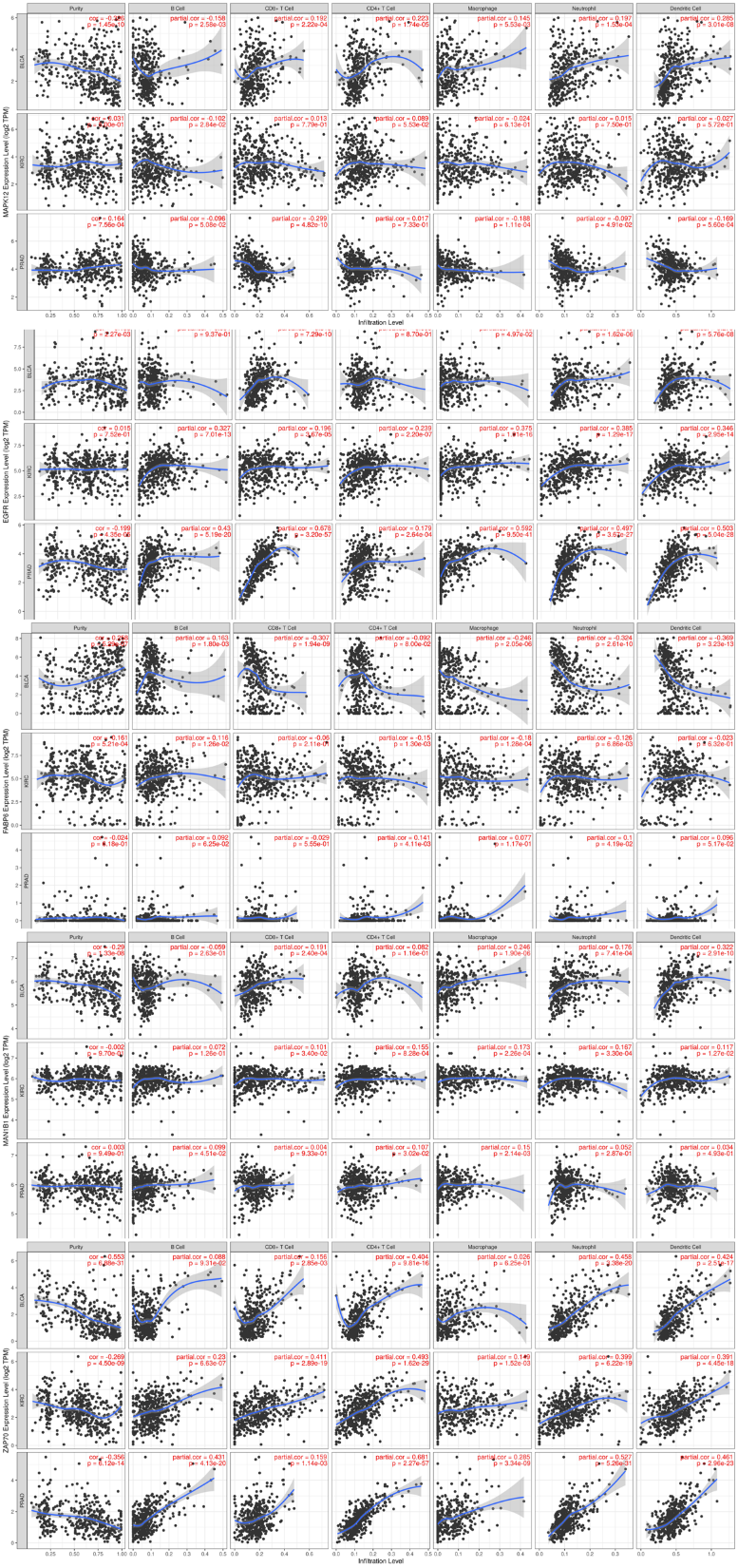


Supplementary Figure 2 The relationship between immune cells and gene expression in urinary tumors
